# Characterization of Dengue Virus 4 Cases in Paraguay, 2019–2020

**DOI:** 10.3390/v16020181

**Published:** 2024-01-25

**Authors:** Alejandra Rojas, John Shen, Fátima Cardozo, Cynthia Bernal, Oliver Caballero, Sara Ping, Autum Key, Ali Haider, Yvalena de Guillén, Patricia Langjahr, Maria Eugenia Acosta, Laura Aria, Laura Mendoza, Malvina Páez, Marta Von-Horoch, Patricia Luraschi, Sandra Cabral, María Cecilia Sánchez, Aurelia Torres, Benjamin A. Pinsky, Anne Piantadosi, Jesse J. Waggoner

**Affiliations:** 1 Instituto de Investigaciones en Ciencias de la Salud, Universidad Nacional de Asunción, San Lorenzo 111241, Paraguay; fati.cardozo@hotmail.com (F.C.); cbernal@iics.una.py (C.B.); oliver.caballero@hotmail.es (O.C.); ivalenaguillen@yahoo.com (Y.d.G.); maruhetter@yahoo.com.mx (M.E.A.); lauraariazaya@yahoo.es (L.A.); lauramendozatorres@gmail.com (L.M.); paezmalvina@yahoo.es (M.P.); 2 Rollins School of Public Health, Emory University, Atlanta, GA 30322, USA; john.shen@alumni.emory.edu; 3 Departamento de Laboratorio de Análisis Clínicos, Hospital Central—Instituto de Previsión Social, Asunción 001531, Paraguay; mcsanche@ips.gov.py (M.C.S.); autorres@ips.gov.py (A.T.); 4 Department of Medicine, Division of Infectious Diseases, Emory University, 1760 Haygood Drive NE, Room E-169, Bay E-1, Atlanta, GA 30322, USA; sara.ping@emory.edu (S.P.); ali.asghar.haider@emory.edu (A.H.); anne.piantadosi@emory.edu (A.P.); 5 Department of Pathology, Emory University, Atlanta, GA 30322, USA; autum.key@emory.edu; 6 Facultad de Ciencias Químicas, Universidad Nacional de Asunción, Campus Universitario, San Lorenzo 111421, Paraguay; plangjahr@gmail.com; 7 Departamento de Epidemiología, Hospital Central—Instituto de Previsión Social, Asunción 001531, Paraguay; martavhv@gmail.com (M.V.-H.); plurasch@ips.gov.py (P.L.); secabral@ips.gov.py (S.C.); 8 Department of Pathology, Stanford University School of Medicine, Stanford, CA 94305, USA; bpinsky@stanford.edu; 9 Department of Medicine, Division of Infectious Diseases and Geographic Medicine, Stanford University School of Medicine, Stanford, CA 94305, USA

**Keywords:** dengue, Paraguay, NS1, rRT-PCR, phylogenetic analysis

## Abstract

In 2019–2020, dengue virus (DENV) type 4 emerged to cause the largest DENV outbreak in Paraguay’s history. This study sought to characterize dengue relative to other acute illness cases and use phylogenetic analysis to understand the outbreak’s origin. Individuals with an acute illness (≤7 days) were enrolled and tested for DENV nonstructural protein 1 (NS1) and viral RNA by real-time RT-PCR. Near-complete genome sequences were obtained from 62 DENV-4 positive samples. From January 2019 to March 2020, 799 participants were enrolled: 253 dengue (14 severe dengue, 5.5%) and 546 other acute illness cases. DENV-4 was detected in 238 dengue cases (94.1%). NS1 detection by rapid test was 52.5% sensitive (53/101) and 96.5% specific (387/401) for dengue compared to rRT-PCR. DENV-4 sequences were grouped into two clades within genotype II. No clustering was observed based on dengue severity, location, or date. Sequences obtained here were most closely related to 2018 DENV-4 sequences from Paraguay, followed by a 2013 sequence from southern Brazil. DENV-4 can result in large outbreaks, including severe cases, and is poorly detected with available rapid diagnostics. Outbreak strains seem to have been circulating in Paraguay and Brazil prior to 2018, highlighting the importance of sustained DENV genomic surveillance.

## 1. Introduction

Dengue virus (DENV) is the most common arbovirus worldwide, with 50–100 million symptomatic infections resulting annually from four related viruses, designated DENV types 1–4 [1]. The reported epidemiology and relative severity of DENV-4 have differed between regions and patient populations, with predominantly secondary cases and less severe disease reported in Southeast Asia compared to a mixture of primary and secondary cases with a spectrum of disease severity in the Americas [2,3,4,5,6,7,8,9,10,11,12,13,14,15]. Five distinct genotypes of DENV-4 have been identified, and genetic differences between genotypes impact both viral biology and neutralization by pre-existing antibodies [16,17]. Genotype II was introduced into the Caribbean in the early 1980s, with multiple subsequent introductions into Brazil from Colombia or Venezuela in the early 2000s and spread to neighboring countries in the Southern Cone [6,18,19,20,21]. While DENV-4 epidemics have been described in the region, with strains emerging/re-emerging from pre-existing lineages [5,6,7,14,22,23,24,25], detection and characterization of dengue cases caused by DENV-4 have been hampered by the poor performance of available rapid diagnostics for this virus type [26,27,28]. The “gold standard” for dengue diagnosis has long been considered the detection of seroconversion between acute and convalescent samples [29,30], however, paired samples are frequently unavailable in clinical practice, further limiting detection by this method.

Paraguay is hyperendemic for DENV, with sustained viral circulation since 1999 and large disease outbreaks occurring every 2–5 years [31]. Dengue occurs throughout the country, but most cases are detected in metropolitan Asunción, which is the most populated area in the country and includes the capital and surrounding Central Department. Typically, a single DENV type predominates during the high transmission season from November through April. However, other types are also detected at lower rates [19,31,32,33] or with regional transmission [34]. DENV-4 was first identified in Paraguay in 2012 and circulated at low levels from 2012 to 2018. From 2015 to 2018, DENV-1 was predominant [31,33], but in 2019–2020, DENV-4 emerged to cause the largest DENV outbreak in the country’s history [31,35].

A previous study of temporal distribution of DENV in Paraguay revealed epidemic waves yearly recurrently during the late summer months. Moreover, the mosquito-viral suitability index accurately corresponded to the seasonal timing of reported dengue cases [36]. From February 2019 to March 2020, a bimodal incidence of suspected dengue cases was observed in Paraguay. The first wave extended from March to June 2019, with a peak in April when 2164 suspected cases were reported [37], and the larger second wave began in October 2019, when an epidemiologic alert for dengue was issued and a sustained increase in suspected cases was reported, with a peak in February 2020 with more than 33,200 suspected cases registered [38].

Published genomic data indicate that 2018 DENV-4 strains were most closely related to strains circulating in southern Brazil, circa 2013 [19,31]. Furthermore, a recent study showed that DENV-4 strains that circulated in Paraguay in 2020 were also related to viruses circulating in midwestern and southwestern Brazil [36]. Despite the recent advances/studies in DENV phylogenetics, more genomic information is required to understand the epidemiologic pattern and virus population dynamics in Paraguay and the neighboring countries. Therefore, the objectives of this study were to (1) describe diagnostic test performance for and clinical manifestations of dengue cases detected in 2019–2020 in Paraguay and (2) perform phylogenetic analyses of identified DENV-4 strains.

## 2. Materials and Methods

### 2.1. Study Participants

Participants of both genders and all ages were enrolled into an ongoing study of suspected arboviral infections between January 2019 and March 2020 from the Hospital Central of the Instituto de Previsión Social or as outpatients at IICS-UNA. Hospital Central, located in Asunción, is a tertiary care hospital that provides medical attention to patients from Asunción, the surrounding metropolitan area, and transfers from throughout the country. IICS-UNA is a research institute in San Lorenzo, which is in metropolitan Asunción, Central Department. Inclusion criteria were an acute illness including two or more of the following symptoms: fever (measured or subjective), red eyes, rash, joint pain involving more than one joint, and/or diffuse muscle pain. Patients with fever and no other localizing signs or symptoms were also included. Day 1 was defined as the day on which symptoms began, and individuals were included in the current study up to 7 days post-symptom onset. Cases were classified according to the 2009 WHO criteria as dengue without warning signs (DWS-), dengue with warning signs (DWS+), and severe dengue (SD) [30].

### 2.2. Clinical Samples and DENV Testing

Serum was obtained at the enrollment visit, aliquoted, and stored at −80 °C. Participants were screened for DENV by testing for the non-structural protein 1 (NS1) antigen and/or DENV RNA in a multiplex rRT-PCR for Zika, chikungunya, and dengue (the ZCD assay) [33,39,40]. NS1 testing was performed at IICS-UNA using the Standard Q Dengue Duo rapid immunochromatographic test (SD Biosensor, Suwon, South Korea) according to manufacturer recommendations. Screening test results, both positive and negative, were confirmed in a DENV type-specific, quantitative rRT-PCR (the DENV multiplex test, DMPT) [41]. The Standard Q Dengue Duo rapid immunochromatographic test also detects anti-DENV IgM and IgG. Results of antibody detection were recorded but not incorporated into the diagnostic algorithm of acute dengue cases.

The ZCD assay and DMPT were performed at both IICS-UNA and Emory University, following shipment of sample aliquots on dry ice. At IICS-UNA, RNA was extracted from 140 µL of serum using the Viral RNA Mini Kit (Qiagen, Germantown, MD, USA) and eluted into 60 µL of buffer, according to manufacturer recommendations. At Emory, total nucleic acid extraction was performed using either (1) an EMAG instrument (bioMérieux, Durham, NC, USA) or (2) the MagMaxViral RNA Isolation Kit in a KingFisher Apex system (both from ThermoFisher Scientific, Waltham, MA, USA). For automated extractions, nucleic acids were extracted from 200 µL of serum and eluted in 60 μL of buffer. A total of 5 μL of eluate was then used in ZCD and DMPT reactions, and both assays were performed and interpreted as previously described [39,40,41]. Serum viral load was quantified from 4-point standard curves prepared with synthesized DENV target sequences and included on dedicated DMPT runs.

### 2.3. Case Definitions

Dengue case confirmation required a positive result in the DMPT. Cases that (1) tested negative for DENV in the ZCD assay or (2) had a positive screening test (NS1 or ZCD) that could not be confirmed in the DMPT were considered other acute illness (OAI). This case definition was employed to ensure rigorous confirmation of dengue cases with at least two different tests.

### 2.4. DENV Sequencing

Sixty-two 2019–2020 samples were selected for sequencing from individuals with confirmed DENV-4 infections and DMPT Ct values < 35. Samples were further selected to represent the distribution of all cases based on month of collection, city of residence, and severity of clinical illness. All samples from SD cases that met the Ct criterion were selected. A single DENV-4 case collected in 2018 as part of this ongoing study was also sequenced and included in phylogenetic analyses [33].

Extracted total nucleic acid underwent heat-labile dsDNase treatment (ArcticZymes, Tromso, Norway). cDNA was synthesized using random hexamer primers and SuperScript III RT (both from ThermoFisher Scientific) for first strand synthesis and New England Biolabs (New England Biolabs, Inc., Ipswich, MA, USA) reagents for second strand synthesis, without amplification. Sequencing libraries were fragmented and indexed using the Nextera XT DNA Library Prep kit (Illumina, San Diego, CA, USA) with dual indexes and 16 cycles of PCR. Libraries were quantified using the KAPA universal complete kit (Roche, Basel, Switzerland), pooled to equimolar concentration, and sequenced on a MiSeq with paired-end 150-bp reads (Illumina, San Diego, CA, USA). As a negative control, water was included with each batch of samples starting from DNase. As a positive control, in vitro transcribed ERCC spike-ins (NIST) were added to each sample prior to cDNA synthesis.

Sequencing reads underwent reference-based assembly using viral-ngs version 2.0.21.3-rc20 (github.com/broadinstitute/viral-pipelines; date accessed, 01 February 2022) and reference sequence KP188564.1. Consensus sequences from each sample were aligned and visually inspected using Geneious R8 (Biomatters, San Francisco, CA, USA). Genotyping was performed using the online Genome Detective Virus Tool (https://www.genomedetective.com; date accessed, 1 February 2022) [42]. Complete DENV-4 genomes were downloaded from the Bacterial and Viral Bioinformatics Resource Center (BV-BRC, https://www.bv-brc.org/; date accessed, 1 February 2022) as reference sequences for phylogenetic analysis. These were MAFFT aligned with our Paraguay DENV-4 sequences using Geneious Prime (Biomatters, Inc., San Diego, CA, USA), and untranslated regions in the 5′ and 3′ ends were trimmed.

Maximum-likelihood (ML) phylogenies were estimated with IQ-TREE (version 1.6.12) with ultrafast bootstrap approximation to evaluate clade probabilities. ModelFinder was used to select the GTR+F+gamma4 nucleotide substitution model [43]. Temporal signal was assessed using TempEst v1.5.1 [44], and 12 reference sequences with >0.01 distance from the best-fitting linear regression were excluded as outliers for possible low sequencing quality or misclassified dates. Downsampling was performed from this alignment to yield a set of unique sequences with high genome coverage of predominantly the same genotype identified in this study (genotype II; see Supplemental Material for complete details). Our final dataset included 61 DENV-4 sequences generated by our group from 2019 to 2020, 1 DENV-4 sequence generated by our group from 2018, 9 reference sequences from Paraguay in 2018, and 129 globally representative DENV-4 genotype II reference sequences. The final ML phylogenetic tree was rooted on the oldest DENV-4 sequence.

Time-scaled phylogenetic trees were constructed in BEAST v1.10.4 using a GTR+gamma4 substitution model with 3 codon positions, a relaxed molecular clock, and 200,000,000 Markov chain Monte Carlo steps [45]. TreeAnnotator v1.10.4 was used to summarize the maximum clade credibility (MCC) tree after 10% burn-in [45]. ML and time-scaled trees were visualized through the interactive Tree of Life v6 (iTOL, https://itol.embl.de; date accessed, 10 February 2022) and FigTree v1.4.4 (http://tree.bio.ed.ac.uk/software/figtree; date accessed, 10 February 2022).

### 2.5. Statistical Analysis

Basic statistical analyses were performed using Excel software version 2312 (Microsoft, Redmond, WA, USA). Comparisons between group means and medians were made by ANOVA, Welch’s test, both pooled and non-pooled two-sample *t*-tests, and Kruskal–Wallis tests. Comparisons of proportions were made using chi-squared tests or Fisher exact tests. Graphs were prepared with GraphPad Prism version 9 (GraphPad, San Diego, CA, USA). Crude associations and statistical analysis were performed using SAS version 9.4. Significance was set at two-sided *p*-values ≤ 0.05 for all analyses.

## 3. Results

### 3.1. Geographical Distribution of Studied Cases

**Figure 1:**
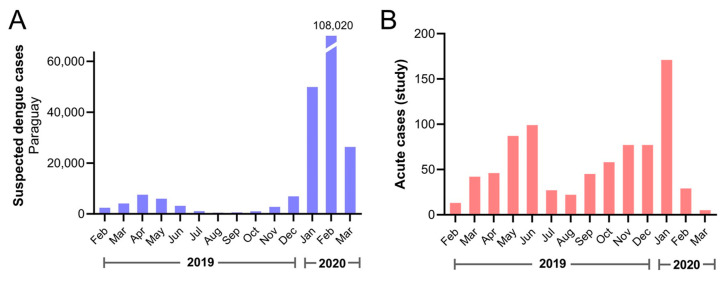
Suspected dengue cases reported in the country and enrolled acute cases during the study period. (**A**) All suspected dengue cases reported to the Ministerio de Salud Pública y Bienestar Social, Paraguay by month. The number of cases for February 2020 is shown above the broken bar. (**B**) Acute cases included in the current study by date of symptom onset.

**Figure 2:**
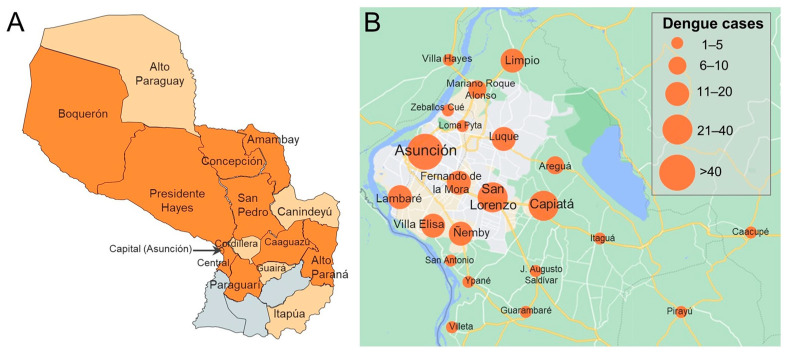
(**A**) Map of Paraguay displaying departments from which dengue cases were enrolled (dark orange) or other acute illness cases were enrolled but dengue was not identified (light orange). Three departments from which no cases were enrolled are shown in grey (from west to east: Ñeembucú, Misiones, Caazapá). (**B**) Map of dengue cases detected in the study by city in the capital district (Asunción) and surrounding area. Maps were prepared using (**A**) Mapchart (www.mapchart.net; date accessed, 23 August 2022) and (**B**) Google Maps, 2022 (www.google.com/maps; date accessed, 23 August 2022).

Participants included in the current study were enrolled between February 2019 and March 2020, and the distribution approximately mirrored country-wide numbers of suspected dengue cases, both confirmed and unconfirmed, reported to the Ministerio de Salud Pública y Bienestar Social, Paraguay (Figure 1). Patients from 14 of 17 departments and the capital district of Paraguay were included (Figure 2A). Most dengue (229/253, 90.5%) and OAI cases (501/546, 91.8%) came from the Central Department or capital district (Table S1). Dengue cases were confirmed among individuals who resided in 9 departments and the capital district (Figure 2).

### 3.2. Study Population

**Figure 3:**
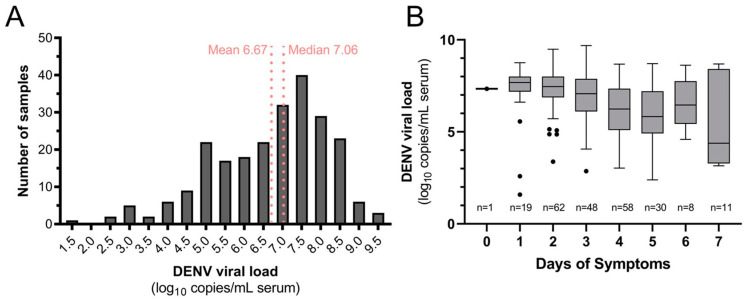
Quantifiable DENV-4 serum viral load (**A**) distribution for dengue cases in the study population and (**B**) by day of symptoms at presentation.

Seven hundred ninety-nine participants were enrolled and met inclusion criteria. This included 253 (31.7%) confirmed dengue and 546 (68.3%) OAI cases (Table 1). Dengue cases were older (mean 36.1 years, standard deviation (SD) 20.1) than OAIs (27.9, SD 19.3; *p* < 0.001) but were similar in gender makeup, comorbid illnesses, and days of symptoms at presentation (Table 1). DENV-4 was identified in 238 cases (94.1%), followed by DENV-2 (14, 5.5%) and DENV-1 (1, 0.4%). No mixed infections were detected, and no Zika or chikungunya cases were detected. DENV-4 serum viral load was quantifiable for 237/238 cases (99.6%, mean 6.67 log_10_ copies/mL, SD 1.50; Figure 3A), and viral load declined overall with days of symptoms (Figure 3B).

DENV NS1 detection by rapid test demonstrated 52.5% sensitivity (53/101) and 96.5% specificity (387/401) compared to rRT-PCR (Table 2A). DENV viral load was not significantly different among samples with detectable versus undetectable NS1 or anti-DENV IgM (Figure S1). Sensitivity of NS1 detection was lowest on days 1 and 2 of symptoms (20–37%), with improved but variable detection from days 3 to 7 (44–76%; Figure S2). Anti-DENV IgM was detected in 25/96 dengue cases (26.0%) and 47/387 OAI cases (12.1%; Table 2B). Anti-DENV IgM detection did not demonstrate a consistent trend across days of symptoms (Figure S2). Of all samples analyzed for antibody detection, anti-DENV IgG was detected in 242/483 samples (50.1%) and 54/96 dengue cases (56.3%; Table 2C).

### 3.3. Clinical Manifestations and Severity

Symptoms reported among dengue and OAI cases are shown in Table 3. After correction for multiple comparisons, arthralgias, myalgias, and nausea remained significantly more common among dengue cases, whereas cough and sore throat were less common. Most participants reported having fever in the preceding 7 days (235/252 dengue (93.3%) and 480/531 OAI (90.4%) cases), and measured temperature did not differ between the groups (dengue, mean 38.7 °C (SD 0.7) and OAI 38.7 °C (0.8)). Of dengue cases, 136 (53.8%) were categorized as DWS-, 103 (40.7%) DWS+, and 14 (5.5%) SD.

The study population included 106 pregnant women: 22 dengue and 84 OAI cases. Two pregnant women (9.1%) had DWS+ (no SD cases). However, 19/22 (86.4%) were hospitalized, which was significantly higher than the proportion of hospitalized pregnant women with OAI cases (18/43 with disposition data (41.9%), *p* < 0.001).

### 3.4. Phylogenetic Analysis

DENV sequences in the final alignment included 138 reference and 62 newly generated sequences from Paraguay: 61 from 2019 to 2020 (Tables S2 and S3, Figure S3) and a single sequence from 2018 (NCBI-GenBank accession numbers: OP811915–OP811976). All newly generated DENV-4 sequences belonged to genotype II. Reference sequences represented samples collected from 1956 to 2018, including sequences from Asia and the Americas (Table S4).

**Figure 4:**
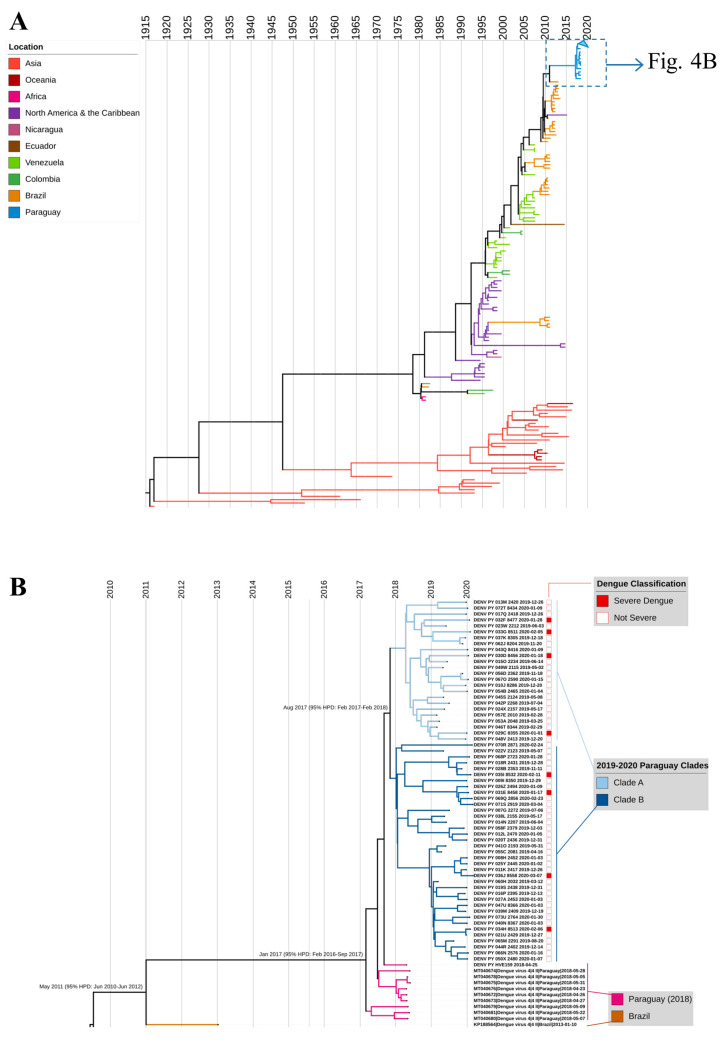
MCC tree of DENV-4 genotype II. (**A**) Full tree with 200 sequences from the final dataset: 61 DENV-4 from Paraguay, 2019–2020 (this study); 1 DENV-4 sequence from Paraguay, 2018 (this study); 9 reference sequences from Paraguay in 2018; and 129 globally representative DENV-4 sequences. (**B**) Magnified and cropped MCC tree to show Paraguay 2019–2020 outbreak sequences and closest references. SD cases are indicated with a solid red box; all other dengue cases are indicated with an empty box. Years are indicated by vertical lines. Nodes for the MRCA are labeled with the month and year (95% confidence interval).

In the ML phylogenetic analysis, all Paraguay sequences clustered together (Figure 4A, demarcated with a dashed line box), and outbreak sequences were most closely related to 2018 sequences from Paraguay, which clustered just basal to the sequences from this study (Figure S4). All ten Paraguay sequences from 2018 differed from the outbreak sequences by only two synonymous mutations, one in the *NS3* gene and the other in *NS5*. The closest reference sequence from outside Paraguay came from a sample collected in São José do Rio Preto, Brazil in 2013 (KP188564.1). Outbreak strains comprised two clades, designated clade A (n = 24) and clade B (n = 37) (Figure 4B, tree branches shown in different shades of blue). Clades A and B differed by three synonymous single nucleotide polymorphisms, one each in the *envelope*, *NS3*, and *NS5* genes. In ML analysis, Clade A appeared to be more closely related to the 2018 DENV-4 sequence generated for the current study, but in Bayesian analysis, that sequence was confirmed as ancestral to both. There was no phylogenetic clustering of cases by severity (Figure 4B), geographic location, or epidemic wave.

In time-scaled phylogenetic analysis, outbreak sequences again clustered together with high support and shared a most recent common ancestor in August 2017 (95% highest posterior density (HPD) February 2017–February 2018; Figure 4). Our inferred mean clock rate of 8.79 × 10^−4^ (95% HPD 7.85–9.80 × 10^−4^) is slightly higher than the median reported rate in prior studies on DENV-4, 7.91 × 10^−4^, but well within the range of reported rates, 6.89 × 10^−4^ to 20 × 10^−4^ [46]. All Paraguay sequences shared a common ancestor in January 2017 (95% HPD February 2016–September 2017) and diverged from their most recent ancestor, KP188564_Brazil_2013, in May 2011 (95% HPD June 2010–June 2012). These results suggest that there was unappreciated circulation of the outbreak lineage between 2011 and 2017. To assess whether the lineage was captured in prior studies of partial genome sequencing, we analyzed 743 DENV-4 reference sequences from the BV-BRC database collected between 2012 and 2018 with at least full *envelope* sequences (1485 bp), and we found no additional closely related reference sequences.

## 4. Discussion

From the end of 2019 to early 2020, DENV-4 caused the largest DENV outbreak in Paraguay’s history [31,35]. In our study population, dengue cases were poorly detected with available rapid diagnostics, associated with certain clinical manifestations, and some progressed to SD. The relative clinical severity of DENV-4 has varied in prior studies, which may reflect differences in virus strains and/or patient populations. In studies from Southeast Asia with documented transmission of all four DENV types, DENV-4 is often the least common, predominantly detected among secondary cases and associated with lower severity than other types [9,10,12]. SD risk with DENV-4 may also be lower in the Americas, particularly compared to DENV-2 [3,4,13,14,15]. However, consistent with our findings, DENV-4 still causes SD, with an overall risk similar to DENV-1 [11,13,15] and increased risk among older patients and those with secondary infections [4,5,6,7,8,9,11,13,47].

Phylogenetic analysis indicated that the DENV-4 lineage responsible for the 2019–2020 outbreak was nearly identical to viruses detected in Paraguay in 2018, consistent with a recent phylogenetic study of DENV in the country [36]. All Paraguay DENV-4 sequences shared a most recent common ancestor in 2017, and this aligns with a molecular clock analysis on 2018 DENV-4 sequences that estimated viral introduction into Paraguay in September 2017 [19]. Thus, this large outbreak was not due to introduction of a new lineage into the country in 2019 but instead resulted from local DENV-4 evolution and emergence in a susceptible population [22,23,25]. While we did not observe fixation of nonsynonymous mutations among DENV-4 sequences from Paraguay, these sequences all differed from their closest ancestor (KP188564_Brazil_2013) by seven amino acids, and the evolutionary history of this lineage over the decade preceding the outbreak is unclear due to limited DENV-4 sequences from the country and region.

DENV-4 has undergone multiple introductions into South America over the past 40 years, and genotype II, as identified in our study, has been predominant [5,6,7,16,18,20,21,24,25,47]. After being absent for three decades, DENV-4 was detected in Brazil in 2010 and resulted in explosive epidemics in the following years, probably because of the population’s susceptibility [5,48]. Similar to findings in Puerto Rico, previous research hypothesized that DENV-4 re-emergence or re-introduction in the state of Roraima in 2010 was preceded by cryptic or imperceptible circulation of the virus [20,49]. Several studies have demonstrated that densely populated states like Sao Paulo and Rio de Janeiro play a key role in the spread of DENV-4 to other Brazilian locations [48,50], and notably, the MRCA for DENV-4 strains in Paraguay was detected in Sao Paulo state. Autochthonous DENV-4 evolution that precedes viral re-emergence and the ongoing risk for introduction of new strains, such as genotype I introduction into Brazil from Asia [20], highlights the importance of sustained genomic monitoring to trace the origin of new outbreaks.

It is notable that DENV-4 emerged in Paraguay in a population where DENV-1 had been predominant for the previous four years [33], as both the change in the predominant DENV type and waning cross-protective immunity could have contributed to the high numbers of symptomatic infections seen in 2019–2020 [51,52]. The DENV-1/DENV-4 order of infections has been observed among SD cases [53], and prior DENV-1 infection has disproportionately contributed to SD elsewhere [54]. The wave dynamics observed in 2019–2020 fit with the arrival of DENV-4 outbreak strains in a susceptible population relatively late in the DENV transmission season that ended in early 2019 in Paraguay [55], and consistent with this, genetic differences were not observed between the two waves. All sequenced SD cases were detected in the second wave. However, these did not cluster in phylogenetic analyses, and this finding may be attributed to higher case numbers in the second wave increasing observed SD by chance.

Rapid NS1 testing demonstrated poor sensitivity (52.5%) for dengue cases caused by DENV-4. This was lower than the sensitivity of the same assay observed during the 2018 DENV-1 outbreak in Asunción (71.4%), though specificity was high (>96%) in both studies [33]. These data are similar to findings from Brazil, where rapid immunochromatographic tests for NS1 resulted in under-detection of DENV-4 [26,27,28,56]. Poor NS1 performance may result from lower levels of NS1 in DENV-4 cases, though data to this effect are sparse [57], or high seroprevalence of anti-DENV IgG. Sensitivity of NS1 detection may improve with heat dissociation of IgG-NS1 complexes [5,56], but this requires instrumentation and detracts from the benefits of point-of-care testing. Due to NS1 test performance, this was implemented only as a screening test to determine further work-up by rRT-PCR. NS1 rapid tests continue to be a widely used tool in clinical practice, particularly in sites with limited resources due to simplicity and relatively low cost [58,59]. However, it is important to consider the potential clinical and epidemiologic impact of their reduced sensitivity in comparison to DENV RNA detection shown in this work, particularly for DENV-4. This emphasizes the necessity of developing point-of-care diagnostic tests with improved performance features [60,61].

The clinical presentation of dengue cases differed from that of OAIs in this population, with arthralgia, myalgia, and nausea reported significantly more often among cases and cough and sore throat reported less often. DENV-4 has previously been associated with cutaneous manifestations when compared to other DENV types [34]. Although rash was also more common among dengue cases in our population, this did not remain significant after adjustment for multiple comparisons. Sore throat was also less common among DENV-1 cases from Asunción in 2018, when dengue cases more commonly experienced headache and conjunctivitis [33]. Although these remain relatively general complaints, identification of such symptom constellations will aid clinicians in the judicious use and interpretation of available diagnostics.

## 5. Limitations

This study focused on acute symptomatic dengue cases. Therefore, results may not be generalizable to mild or subclinical DENV-4 infections, and primary/secondary infection status could not be fully characterized. Second, although participants resided in 14/17 departments in Paraguay, over 90% of individuals lived in Asunción or the Central Department, which impacts the power of phylogenetic studies to detect regional differences in DENV-4 sequences. Third, the genomic record for DENV-4 in Paraguay dates back only to 2018 and is limited in South America as a whole. This complicated analyses of DENV-4 introduction into Paraguay and the emergence of the two clades identified in the current outbreak. Nevertheless, this study provides important information on a large dengue epidemic that occurred in an endemic country like Paraguay and could serve to improve our understanding of dengue epidemiology in the region.

## 6. Conclusions

Findings from the 2019–2020 DENV outbreak in Paraguay highlight the capacity of DENV-4 to cause explosive outbreaks and the need for sustained genomic monitoring of circulating DENV strains in a population. DENV-4 is poorly detected with available rapid diagnostics and, without high rates of symptomatic disease and widespread molecular testing, may remain under-reported and insufficiently characterized.

## Figures and Tables

**Table 1:** Demographic and clinical data for dengue cases versus other acute illness cases (N = 799).

Characteristic	Dengue ^a^ n = 253	Other Acute Illness ^a^ n = 546	*p*-Value
Age, years, mean (SD)	36.1 (20.1)	27.9 (19.3)	<0.001
Gender, female	158 (62.5)	348 (63.7)	0.75
Comorbidity, ≥1	73 (28.9)	141 (25.8)	0.39
Day of symptoms, mean (SD)	3.39 (1.6)	3.38 (1.6)	0.92
DENV type			
DENV-1	1 (0.4%)	–	–
DENV-2	14 (5.5%)	–	–
DENV-4	238 (94.1%)	–	–

Abbreviation: SD, standard deviation. ^a^ Data presented as n/N (%).

**Table 2:** Comparison of (A) NS1, (B) IgM, and (C) IgG detection in acute dengue cases confirmed by rRT-PCR.

**A**		**DENV rRT-PCR**		
		**Positive**	**Negative**	**Total**
**NS1**	**Positive**	53	14	67
	**Negative**	48	387	435
	**Total**	101	401	**502**
**B**		**DENV rRT-PCR**		
		**Positive**	**Negative**	**Total**
**Anti-DENV IgM**	**Positive**	25	47	72
	**Negative**	71	340	411
	**Total**	96	387	**483**
**C**		**DENV rRT-PCR**		
		**Positive**	**Negative**	**Total**
**Anti-DENV IgG**	**Positive**	54	188	242
	**Negative**	42	199	241
	**Total**	96	387	**483**

**Table 3:** Symptoms reported in the preceding 7 days among dengue and other acute illness cases.

Characteristic	Dengue ^a^	Other Acute Illness ^a^	*p*-Value ^b^
Fever	235/252 (93.3%)	480/531 (90.4%)	0.22
Conjunctivitis	88/246 (35.8%)	147/526 (27.9%)	0.029
Rash	72/247 (29.1%)	105/526 (20.0%)	0.006
**Arthralgia**	**199/243 (81.9%)**	**325/510 (63.7%)**	**<0.001**
**Myalgia**	**209/243 (86.0%)**	**372/511 (72.8%)**	**<0.001**
Headache	205/247 (83.0%)	406/522 (77.8%)	0.10
Lethargy	137/247 (55.5%)	261/522 (50.0%)	0.16
Retro-ocular pain	123/247 (49.8%)	203/522 (38.9%)	0.005
**Cough**	**27/247 (10.9%)**	**157/522 (30.1%)**	**<0.001**
Difficulty breathing	52/247 (21.0%)	101/522 (19.3%)	0.63
Back pain	103/247 (41.7%)	171/522 (32.8%)	0.019
**Sore throat**	**30/247 (12.1%)**	**134/522 (25.7%)**	**<0.001**
Abdominal pain	87/247 (35.2%)	142/522 (27.2%)	0.028
**Nausea**	**116/247 (47.0%)**	**184/522 (35.2%)**	**0.002**
Vomiting	56/247 (22.7%)	168/522 (32.2%)	0.007
Diarrhea	42/247 (17.0%)	84/522 (16.1%)	0.76
Edema	10/247 (4.0%)	26/522 (5.0%)	0.72
Bleeding	20/247 (8.1%)	35/523 (6.7%)	0.55
Itching	27/247 (10.9%)	43/522 (8.2%)	0.23

^a^ Results presented as n/N (%): the number of participants who reported a symptom (n) over the total number with recorded data for that symptom field (N) and percent. ^b^ Bold indicates a significant difference in the proportion of dengue and other acute illness cases reporting a symptom using a Bonferroni-corrected *p*-value for significance of ≤0.0026.

## Data Availability

Upon publication, data supporting the presented results will be made freely available through the Emory Dataverse, which is an open data repository offered through a partnership between Emory and the Odum Institute at the University of North Carolina at Chapel Hill.

## Supplementary Materials

The following supporting information can be downloaded at: https://www.mdpi.com/article/10.3390/v16020181/s1, Supplemental methods. Downsampling of DENV-4 sequences. Figure S1. DENV viral load vs. (A) NS1 antigen or (B) anti-DENV IgM. Figure S2. NS1 and anti-DENV IgM rapid diagnostic test performance by day post-symptom onset. Figure S3. Epidemiologic week of symptom onset for 61 sequenced DENV-4 samples. Figure S4. ML trees of DENV-4 genotype II. Table S1. Department or district of residence of dengue and other acute illness cases. Table S2. Data for DENV-4 samples sequenced in this study. Table S3. Demographic data for participants from whom DENV-4 whole genome sequences were obtained. Table S4. Sequences retrieved from NCBI GenBank included for the phylogenetic analysis.
