# Supplementary material for: Characterization of Dengue Virus 4 Cases in Paraguay, 2019–2020
Source: Viruses. 2024 Jan 25;16(2):181. doi: 10.3390/v16020181 (PMC10892180; doi:10.3390/v16020181)
Supplement: Supplementary file 1 [file viruses-16-00181-s001.zip › viruses-2711288-supplementary.pdf]

Characterization of Dengue Virus 4 Cases in Paraguay, 2019–2020

Supplementary Material

Alejandra Rojas, John Shen, Fátima Cardozo, Cynthia Bernal, Oliver Caballero, Sara Ping, Autum Key, Ali Haider, Yvalena de Guillén, Patricia Langjahr, Maria Eugenia Acosta, Laura Aria, Laura Mendoza, Malvina Páez, Marta Von-Horoch, Patricia Luraschi, Sandra Cabral, María Cecilia Sánchez, Aurelia Torres, Benjamin A. Pinsky, Anne Piantadosi and Jesse J. Waggoner

Supplemental Methods

Downsampling of DENV-4 sequences. The following steps were taken to downsample the set of 628 DENV-4 reference sequences from ViPR used in phylogenetic analysis, while maintaining representation across geography and time. Seventeen sequences, excepting sequences from Paraguay, were removed due to low coverage (>10% of the genome contained ambiguous or missing bases). Four sequences were removed because they were collected after the study period. Twenty-eight specimens with identical sequences, year of collection, and country of origin to another specimen were removed. Since the Paraguay DENV-4 sequences were most closely related to genotype II, 365 references sequences from the other three genotypes (4-I, 4-III, 4-IV sylvatic) were removed based on the ViPR genotyping/subtyping tool (ViPR, 2022). Fourteen sequences were removed because either year or country of collection was missing. Finally, our downsampling scheme restricted sequences to one per year per country for reference samples outside of the Americas, ten per year per country within the Americas but excluding Paraguay, and all Paraguay samples. At the beginning of an exploratory maximum likelihood (ML) analysis, a composition chi-square test was performed by IQ-TREE for every sequence to test for homogeneity of character composition (Trifinopoulos et al., 2016). One sequence, MT040677 (Paraguay, 2018), failed this composition test because it significantly deviated from the mean composition in the alignment and depicted an unusually long branch topology in the resulting tree. Thus, this sequence was considered misclassified and removed.

Figure S1. DENV viral load did not differ significantly among individuals with and without detectable (A) NS1 antigen or (B) anti-DENV IgM in the Standard Q Dengue Duo rapid test. Abbreviations: Pos, Positive; Neg, Negative; Ns, non-significant.

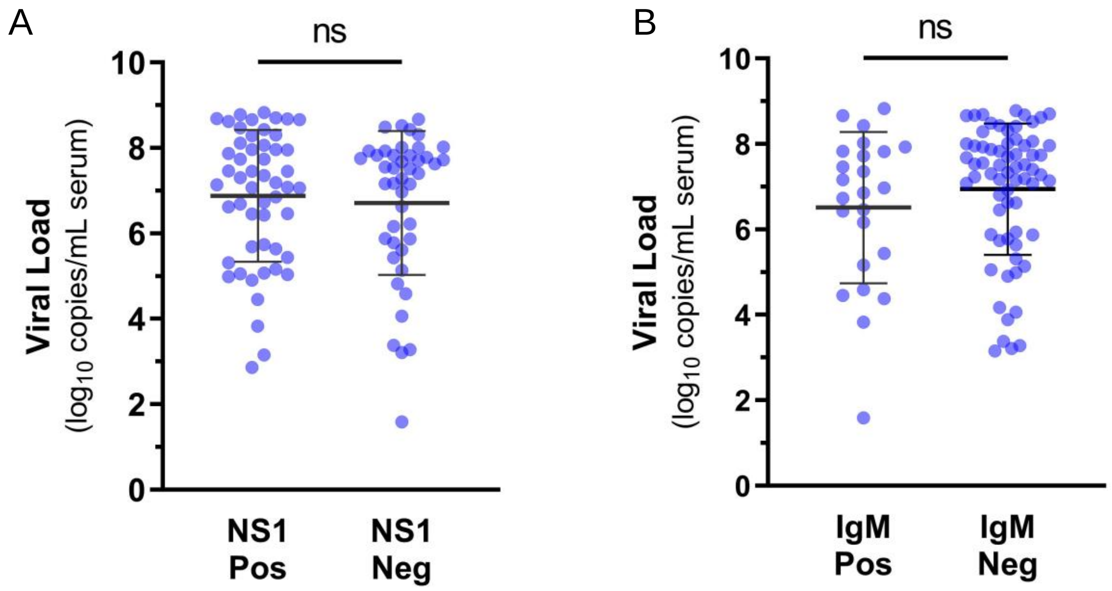

Figure S2. NS1 and anti-DENV IgM rapid diagnostic test performance by day post-symptom onset. Dashed lines show average sensitivity across the first seven days post-symptom onset.

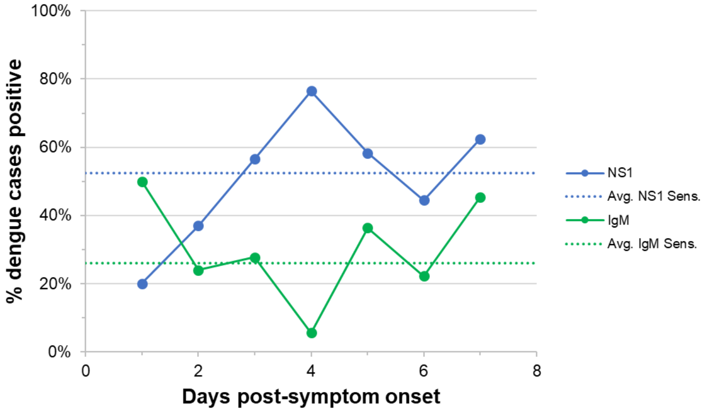

Day	1	2	3	4	5	6	7
NS1, n	5	27	23	17	12	9	8
IgM, n	4	25	18	18	11	9	11

Figure S3. Epidemiologic week of symptom onset for 61 sequenced DENV-4 samples. Alignment of bars, vertical gridlines, and axis labels was made based on Monday weeks. Dates of symptom onset range from 27 February 2019 to 04 March 2020.

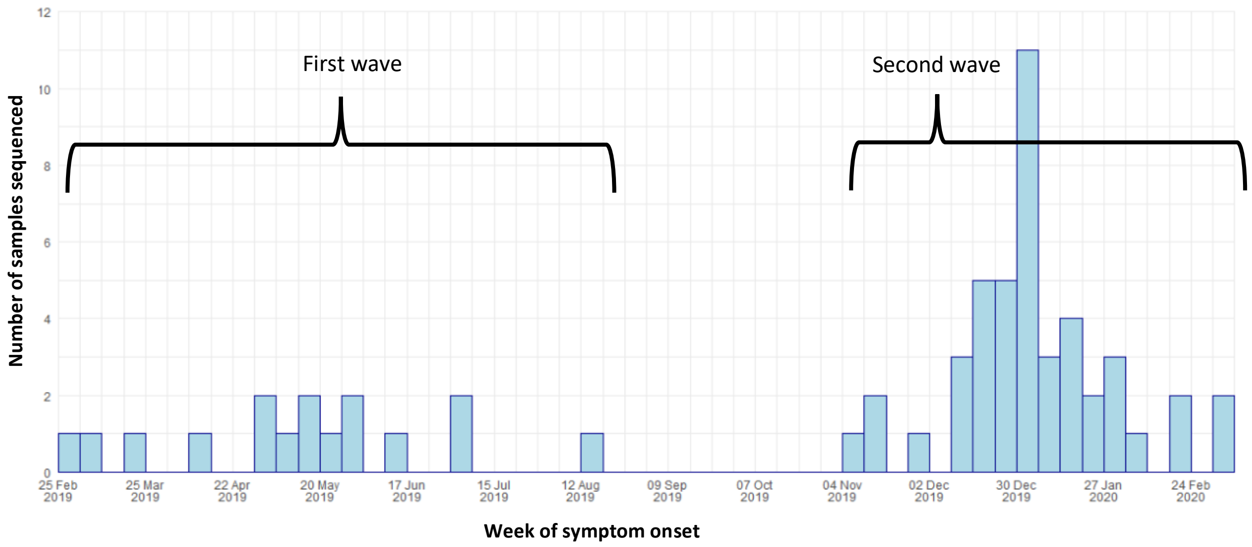

Figure S4. ML trees of DENV-4 genotype II. **A**) Full tree with 200 sequences from the final dataset: 61 DENV-4 from Paraguay, 2019-2020 (this study); one DENV-4 sequence from Paraguay, 2018 (this study); 9 reference sequences from Paraguay in 2018; and 129 globally representative DENV-4 sequences. **B**) Magnified and cropped tree to show Paraguay 2019-2020 sequences and closest references.

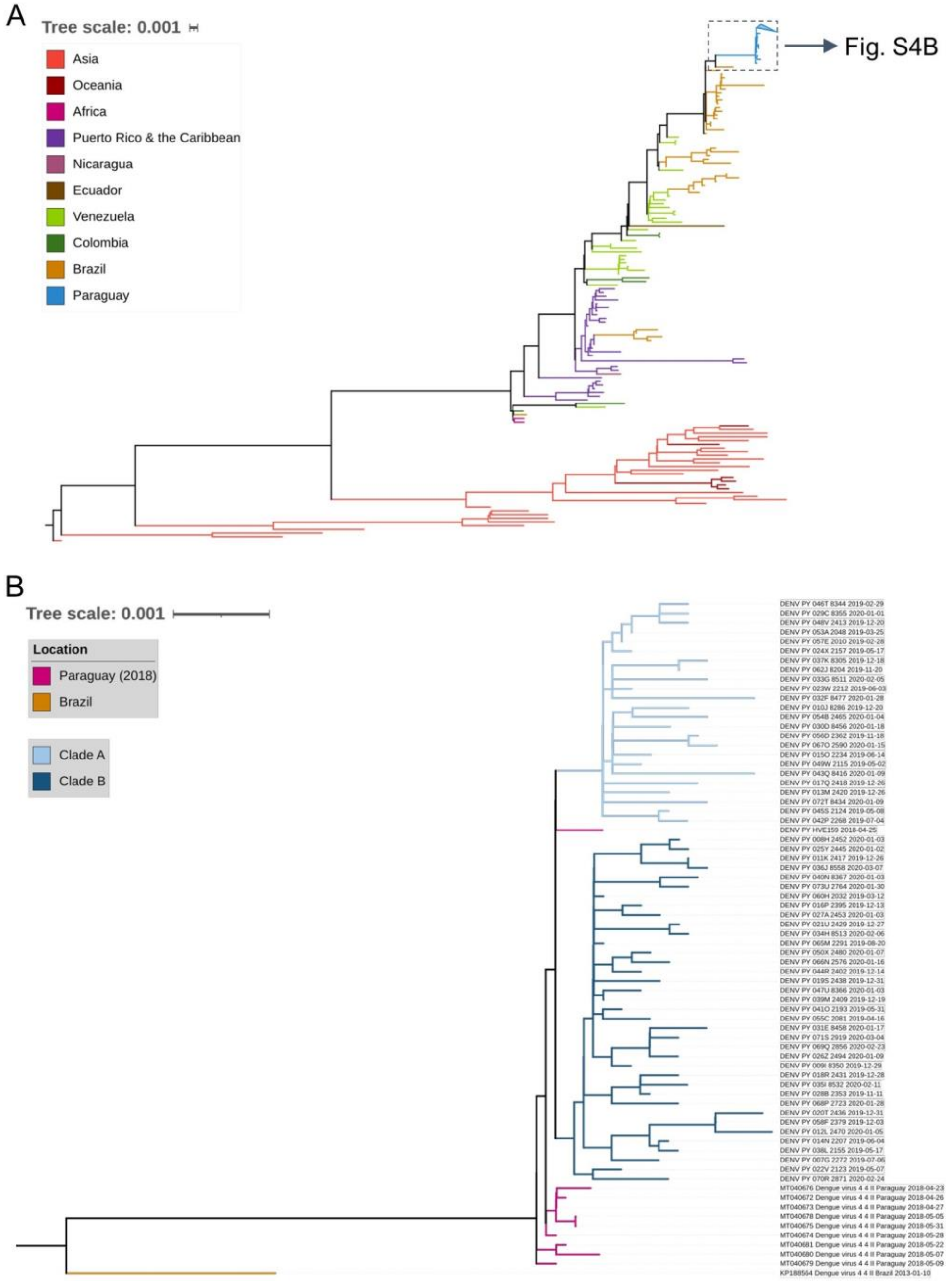

Table S1. Department or district of residence of dengue and other acute illness cases.

**Department/District**	**Dengue cases**[a] n=253	**Other acute illness cases**[a] n=546
Alto Paraguay	0 (0)	1 (0.2)
Alto Paraná	2 (0.8)	1 (0.2)
Amambay	5 (2.0)	2 (0.4)
Boquerón	2 (0.8)	0 (0)
Caaguazú	1 (0.4)	2 (0.4)
Canindeyú	0 (0)	6 (1.1)
Capital (Asunción)	56 (22.1)	135 (24.7)
Central	173 (68.4)	366 (67.0)
Concepción	2 (0.8)	0 (0)
Cordillera	1 (0.4)	12 (2.2)
Guairá	0 (0)	3 (0.5)
Itapúa	0 (0)	1 (0.2)
Paraguarí	6 (2.4)	4 (0.7)
Presidente Hayes	2 (0.8)	8 (1.5)
San Pedro	3 (1.2)	5 (0.9)

[a] Presented as n (%).

Table S2. Data on DENV-4 samples sequenced in this study.

NCBI/GenBank Accession number	Collection Date	City	Department	Genome coverage (%)	Average coverage depth	DMPT Ct value	Serum viral load[a]
OP811915	18-Jan-2020	Pirayú	Paraguarí	95	12	35.62	4.17
OP811916	14-Dec-2019	Areguá	Central	100	1,607	24.31	7.33
OP811917	8-May-2019	Areguá	Central	100	1,403	24.42	7.74
OP811918	29-Dec-2019	Asunción	Capital	100	3,051	24.67	7.33
OP811919	03-Jan-2020	San Lorenzo	Central	100	3,621	24.77	7.30
OP811920	6-Jul-2019	Villeta	Central	100	1.118	24.39	7.68
OP811921	3-Mar-2020	Asunción	Capital	100	9,042	26.51	7.13
OP811922	29-Dec-2019	Yaguarón	Paraguarí	100	25	33.99	4.82
OP811923	20-Dec-2019	Asunción	Capital	100	2,313	23.87	7.55
OP811924	26-Dec-2019	San Antonio	Central	100	957	24.78	7.21
OP811925	05-Jan-2020	Ñemby	Central	100	9,460	27.41	6.89
OP811926	26-Dec-2019	Lambaré	Central	100	1,574	32.23	5.61
OP811927	4-Jun-2019	Asunción	Capital	100	1,974	24.13	7.74
OP811928	14-Jun-2019	Asunción	Capital	100	308	24.47	7.40
OP811929	13-Dec-2019	Fernando de la Mora	Central	100	68	25.53	7.01
OP811930	26-Dec-2019	J. Agusto Zaldívar	Central	100	85	25.75	6.96
OP811931	28-Dec-2019	Capiatá	Central	100	173	32.64	5.50
OP811932	21-Dec-2019	Limpio	Central	100	4,523	27.15	6.96
OP811933	31-Dec-2019	San Lorenzo	Central	100	3,649	26.26	7.20
OP811934	27-Dec-2019	Filadelfia	Boquerón	100	851	25.77	7.33
OP811935	7-May-2019	Ciudad del Este	Alto Paraná	100	1,104	26.65	7.28
OP811936	3-Jun-2019	Puerto Casado	Alto Paraguay	100	40	33.16	5.07
OP811937	17-May-2019	Ñemby	Central	100	6,286	25.08	7.51
OP811938	02-Jan-2020	Villa Elisa	Central	100	26	25.12	7.50
OP811939	09-Jan-2020	Asunción	Capital	100	10,478	26.01	7.50
OP811940	03-Jan-2020	Asunción	Capital	100	3,886	29.00	6.62
OP811941	11-Nov-2019	Lambaré	Central	100	1,628	27.66	6.46
OP811942	01-Jan-2020	Mariano Roque Alonso	Central	100	5,759	23.75	7.50
OP811943	17-Jan-2020	Paraguarí	Paraguarí	100	1,445	30.44	5.63
OP811944	28-Jan-2020	Asunción	Capital	100	2,575	24.36	7.33
OP811945	5-Feb-2020	Luque	Central	100	1,846	24.72	7.23
OP811946	6-Feb-2020	Mariscal Estigarribia	Boquerón	100	6,835	20.51	8.41
OP811947	11-Feb-2020	Areguá	Central	100	737	26.24	6.80
OP811948	7-Mar-2020	No data available	Central	100	2,685	25.30	7.07
OP811949	18-Dec-2019	Itaguá	Central	100	14,101	19.44	8.75
OP811950	17-May-2019	Asunción	Capital	100	9,204	20.29	8.66
OP811951	19-Dec-2019	San Lorenzo	Central	100	6,595	20.69	8.28
OP811952	03-Jan-2020	Capiatá	Central	100	12,243	22.58	7.90
OP811953	31-May-2019	Luque	Central	100	6,268	23.36	7.93

OP811954	4-Jul-2019	Mariano Roque Alonso	Central	100	4,574	24.18	7.75
OP811955	09-Jan-2020	Luque	Central	100	2,233	24.26	7.78
OP811956	20-Dec-2019	Ñemby	Central	100	623	24.90	7.18
OP811957	2-May-2019	Capiatá	Central	100	1,672	25.37	7.53
OP811958	07-Jan-2020	Asunción	Capital	100	3,954	26.25	7.36
OP811959	25-Mar-2019	Villa Elisa	Central	100	1,684	23.52	7.95
OP811960	04-Jan-2020	Asunción	Capital	100	13,705	23.63	7.90
OP811961	16-Apr-2019	Limpio	Central	100	3,508	24.05	7.83
OP811962	18-Nov-2019	Asunción	Capital	100	1,919	24.44	7.30
OP811963	28-Feb-2019	Luque	Central	100	1,725	24.75	7.67
OP811964	03-Dec-2019	Ñemby	Central	100	692	25.87	6.98
OP811965	12-Mar-2019	Capiatá	Central	100	1,532	26.24	7.32
OP811966	20-Nov-2019	Itaguá	Central	100	96	30.06	5.87
OP811967	20-Aug-2019	San Lorenzo	Central	100	232	28.36	6.73
OP811968	16-Jan-2020	Areguá	Central	100	392	27.90	6.50
OP811969	15-Jan-2020	Capiatá	Central	100	2,329	25.49	7.16
OP811970	28-Jan-2020	Asunción	Capital	100	722	28.36	6.38
OP811971	23-Feb-2020	Guarambaré	Central	100	8,524	25.61	7.13
OP811972	24-Feb-2020	San Lorenzo	Central	100	785	28.51	6.27
OP811973	4-Mar-2020	Loma Pyta	Central	100	3,406	25.43	7.18
OP811974	09-Jan-2020	Ñemby	Central	100	1,141	27.25	6.61
OP811975	30-Jan-2020	Asunción	Capital	100	1,016	28.50	6.27
OP811976	25-Apr-2018	Lambaré	Central	100	120	33.38	5.51
-	-	-	-	15	-	38.16	3.38

[a] Expressed as log10 copies/mL of serum

[b] Eliminated from sequence analyses due to insufficient genome coverage

Table S3. Demographic data on 61 participants from whom DENV-4 whole genome sequences were obtained.

**Characteristic**	**Overall**<br>n=61	**DWS-/DWS+**<br>n=53	**Severe dengue**<br>n=8
Age, years, mean (SD)	39.2 (20.4)	35.9 (19.2)	60.9 (15.0)
Gender, female, n (%)	40 (65.6)	36 (67.9)	4 (50.0)

Abbreviations: DWS-, dengue without warning signs; DWS+, dengue with warning signs; SD, standard deviation.

Table S4. Sequences retrieved from NCBI GenBank included for the phylogenetic analysis.

NCBI/GenBank Accession number	Sequence ID	Country/ Location	Date of collection
JN559740	JN559740|Dengue_virus_4|4_II|Brazil|1982-03-26	Brazil	1982-03-26
JQ513334	JQ513334|Dengue_virus_4|4_II|Brazil|2010-11-10	Brazil	2010-11-10
JQ513335	JQ513335|Dengue_virus_4|4_II|Brazil|2011-01-12	Brazil	2011-01-12
JQ513336	JQ513336|Dengue_virus_4|4_II|Brazil|2011-01-11	Brazil	2011-01-11
JQ513337	JQ513337|Dengue_virus_4|4_II|Brazil|2011-01-20	Brazil	2011-01-20
KJ596665	KJ596665|Dengue_virus_4|4_II|Brazil|2012-03-20	Brazil	2012-03-20
KJ579246	KJ579246|Dengue_virus_4|4_II|Brazil|2012-04-19	Brazil	2012-04-19
KJ596659	KJ596659|Dengue_virus_4|4_II|Brazil|2012-03-30	Brazil	2012-03-30
KJ579242	KJ579242|Dengue_virus_4|4_II|Brazil|2012-04-18	Brazil	2012-04-18
JN983813	JN983813|Dengue_virus_4|4_II|Brazil|2010-09-08	Brazil	2010-09-08
MK514144	MK514144|Dengue_virus_4|4_II|Haiti|2015-01-28	Brazil	2015-01-28
JN559741	JN559741|Dengue_virus_4|4_II|Brazil|2010-07-21	Brazil	2010-07-21
KJ596674	KJ596674|Dengue_virus_4|4_II|Brazil|2012-02-16	Brazil	2012-02-16
KJ579245	KJ579245|Dengue_virus_4|4_II|Brazil|2012-03-30	Brazil	2012-03-30
KJ579248	KJ579248|Dengue_virus_4|4_II|Brazil|2012-04-19	Brazil	2012-04-19
KJ579247	KJ579247|Dengue_virus_4|4_II|Brazil|2012-04-12	Brazil	2012-04-12
KU513441	KU513441|Dengue_virus_4|4_II|Brazil|2013-07-02	Brazil	2013-07-02
KP188562	KP188562|Dengue_virus_4|4_II|Brazil|2012-12-10	Brazil	2012-12-10
KP188566	KP188566|Dengue_virus_4|4_II|Brazil|2013-02-08	Brazil	2013-02-08
KP188563	KP188563|Dengue_virus_4|4_II|Brazil|2013-01-07	Brazil	2013-01-07
KT794007	KT794007|Dengue_virus_4|4_II|Brazil|2011-03-07	Brazil	2011-03-07
KP188561	KP188561|Dengue_virus_4|4_II|Brazil|2012-07-11	Brazil	2012-07-11
KP188564	KP188564|Dengue_virus_4|4_II|Brazil|2013-01-10	Brazil	2013-01-10
JQ513330	JQ513330|Dengue_virus_4|4_II|Brazil|2010-07-17	Brazil	2010-07-17
JQ513331	JQ513331|Dengue_virus_4|4_II|Brazil|2010-07-18	Brazil	2010-07-18
JQ513332	JQ513332|Dengue_virus_4|4_II|Brazil|2010-08-20	Brazil	2010-08-20
JQ513333	JQ513333|Dengue_virus_4|4_II|Brazil|2010-08-24	Brazil	2010-08-24

JQ513341	JQ513341|Dengue_virus_4|4_II|Brazil|2010-11-21	Brazil	2010-11-21
JQ513340	JQ513340|Dengue_virus_4|4_II|Brazil|2010-11-29	Brazil	2010-11-29
JQ513338	JQ513338|Dengue_virus_4|4_II|Brazil|2011-01-14	Brazil	2011-01-14
JQ513339	JQ513339|Dengue_virus_4|4_II|Brazil|2011-01-24	Brazil	2011-01-24
JQ513342	JQ513342|Dengue_virus_4|4_II|Brazil|2011-01-29	Brazil	2011-01-29
JQ513343	JQ513343|Dengue_virus_4|4_II|Brazil|2011-01-29	Brazil	2011-01-29
JQ513344	JQ513344|Dengue_virus_4|4_II|Brazil|2011-01-13	Brazil	2011-01-13
KP188565	KP188565|Dengue_virus_4|4_II|Brazil|2013-01-30	Brazil	2013-01-30
FJ196850	FJ196850|Dengue_virus_4|NA|China|1990-07-02	China	1990-07-02
MN018390	MN018390|Dengue_virus_4|NA|China|2013-08-12	China	2013-08-12
KC333651	KC333651|Dengue_virus_4|4_II|China|2012-07-02	China	2012-07-02
JF741967	JF741967|Dengue_virus_4|4_II|China|2010-09-26	China	2010-09-26
GU289913	GU289913|Dengue_virus_4|4_II|Colombia|1982-07-02	Colombia	1982-07-02
GQ868583	GQ868583|Dengue_virus_4|4_II|Colombia|2004-07-02	Colombia	2004-07-02
GQ868584	GQ868584|Dengue_virus_4|4_II|Colombia|2004-07-02	Colombia	2004-07-02
GQ868585	GQ868585|Dengue_virus_4|4_II|Colombia|2005-07-02	Colombia	2005-07-02
GQ868579	GQ868579|Dengue_virus_4|4_II|Colombia|2001-07-02	Colombia	2001-07-02
GQ868580	GQ868580|Dengue_virus_4|4_II|Colombia|2001-07-02	Colombia	2001-07-02
FJ024476	FJ024476|Dengue_virus_4|4_II|Colombia|1997-07-02	Colombia	1997-07-02
MW945720	MW945720|Dengue_virus_4|4_II|Dominica|1981-07-02	Dominica	1981-07-02
KY474335	KY474335|Dengue_virus_4|4_II|Ecuador|2014-07-02	Ecuador	2014-07-02
JQ915084	JQ915084|Dengue_virus_4|4_II|French_Polynesia|2010-06-15	French Polynesia	2010-06-15
JQ915081	JQ915081|Dengue_virus_4|4_II|French_Polynesia|2009-01-29	French Polynesia	2009-01-29
JF262782	JF262782|Dengue_virus_4|4_II|Haiti|1994-07-02	Haití	1994-07-02
KT276273	KT276273|Dengue_virus_4|4_II|Haiti|2014-09-08	Haití	2014-09-08
KP140942	KP140942|Dengue_virus_4|4_II|Haiti|2014-09-11	Haití	2014-09-11
JQ922560	JQ922560|Dengue_virus_4|4|India|2009-07-02	India	2009-07-02
KX845005	KX845005|Dengue_virus_4|4|India|2015-07-02	India	2015-07-02
KU509287	KU509287|Dengue_virus_4|4|India|2009-07-02	India	2009-07-02
KC762696	KC762696|Dengue_virus_4|4_II|Indonesia|2007-08-13	Indonesia	2007-08-13
KC762698	KC762698|Dengue_virus_4|4_II|Indonesia|2008-04-30	Indonesia	2008-04-30

KU523872	KU523872|Dengue_virus_4|4_II|Indonesia|2015-04-08	Indonesia	2015-04-08
KC762697	KC762697|Dengue_virus_4|4_II|Indonesia|2007-12-11	Indonesia	2007-12-11
KU509288	KU509288|Dengue_virus_4|4_II|Indonesia|2010-07-02	Indonesia	2010-07-02
MW945636	MW945636|Dengue_virus_4|4_II|Indonesia|1973-07-02	Indonesia	1973-07-02
MH823210	MH823210|Dengue_virus_4|4_II|Indonesia|2014-07-02	Indonesia	2014-07-02
LC069810	LC069810|Dengue_virus_4|4_II|Japan|2015-07-02	Japan	2015-07-02
MH888334	MH888334|Dengue_virus_4|4_II|Malaysia|2013-01-04	Malaysia	2013-01-04
MH051734	MH051734|Dengue_virus_4|4_II|Malaysia|2014-12-15	Malaysia	2014-12-15
JQ915085	JQ915085|Dengue_virus_4|4_II|New_Caledonia|2008-02-20	New Caledonia	2008-02-20
JQ915086	JQ915086|Dengue_virus_4|4_II|New_Caledonia|2009-02-05	New Caledonia	2009-02-05
MW881266	MW881266|Dengue_virus_4|4_II|Nicaragua|1999-07-02	Nicaragua	1999-07-02
KF041260	KF041260|Dengue_virus_4|4|Pakistan|2009-07-02	Pakistan	2009-07-02
MH382789	MH382789|Dengue_virus_4|4_II|Papua_New_Guinea|2016-07-02	Papua New Guinea	2016-07-02
MT040676	MT040676|Dengue_virus_4|4_II|Paraguay|2018-04-23	Paraguay	2018-04-23
MT040672	MT040672|Dengue_virus_4|4_II|Paraguay|2018-04-26	Paraguay	2018-04-26
MT040673	MT040673|Dengue_virus_4|4_II|Paraguay|2018-04-27	Paraguay	2018-04-27
MT040674	MT040674|Dengue_virus_4|4_II|Paraguay|2018-05-28	Paraguay	2018-05-28
MT040678	MT040678|Dengue_virus_4|4_II|Paraguay|2018-05-05	Paraguay	2018-05-05
MT040675	MT040675|Dengue_virus_4|4_II|Paraguay|2018-05-31	Paraguay	2018-05-31
MT040679	MT040679|Dengue_virus_4|4_II|Paraguay|2018-05-09	Paraguay	2018-05-09
MT040681	MT040681|Dengue_virus_4|4_II|Paraguay|2018-05-22	Paraguay	2018-05-22
MT040680	MT040680|Dengue_virus_4|4_II|Paraguay|2018-05-07	Paraguay	2018-05-07
KY670635	KY670635|Dengue_virus_4|NA|Philippines|2003-10-15	Philippines	2003-10-15
KR011349	KR011349|Dengue_virus_4|4|Philippines|1956-08-28	Philippines	1956-08-28
KU523871	KU523871|Dengue_virus_4|4_II|Philippines|2014-02-13	Philippines	2014-02-13
FJ226067	FJ226067|Dengue_virus_4|4_II|Puerto Rico|1994-07-02	Puerto Rico	1994-07-02
GQ199879	GQ199879|Dengue_virus_4|4_II|Puerto Rico|1994-07-02	Puerto Rico	1994-07-02
GQ199880	GQ199880|Dengue_virus_4|4_II|Puerto Rico|1995-07-02	Puerto Rico	1995-07-02
FJ810417	FJ810417|Dengue_virus_4|4_II|Puerto Rico|1995-07-02	Puerto Rico	1995-07-02
GQ252675	GQ252675|Dengue_virus_4|4_II|Puerto Rico|1995-07-02	Puerto Rico	1995-07-02
FJ850057	FJ850057|Dengue_virus_4|4_II|Puerto Rico|1995-07-02	Puerto Rico	1995-07-02

EU854296	EU854296|Dengue_virus_4|4_II|Puerto Rico|1998-07-02	Puerto Rico	1998-07-02
FJ850059	FJ850059|Dengue_virus_4|4_II|Puerto Rico|1998-07-02	Puerto Rico	1998-07-02
MW945437	MW945437|Dengue_virus_4|4_II|Puerto_Rico|1999-07-02	Puerto Rico	1999-07-02
FJ882600	FJ882600|Dengue_virus_4|4_II|Puerto Rico|1999-07-02	Puerto Rico	1999-07-02
MW945436	MW945436|Dengue_virus_4|4_II|Puerto_Rico|1998-07-02	Puerto Rico	1998-07-02
GQ199885	GQ199885|Dengue_virus_4|4_II|Puerto Rico|1996-07-02	Puerto Rico	1996-07-02
FJ850058	FJ850058|Dengue_virus_4|4_II|Puerto Rico|1996-07-02	Puerto Rico	1996-07-02
FJ882598	FJ882598|Dengue_virus_4|4_II|Puerto Rico|1998-07-02	Puerto Rico	1998-07-02
FJ882597	FJ882597|Dengue_virus_4|4_II|Puerto Rico|1998-07-02	Puerto Rico	1998-07-02
GQ199878	GQ199878|Dengue_virus_4|4_II|Puerto Rico|1994-07-02	Puerto Rico	1994-07-02
GQ199881	GQ199881|Dengue_virus_4|4_II|Puerto Rico|1996-07-02	Puerto Rico	1996-07-02
GQ199883	GQ199883|Dengue_virus_4|4_II|Puerto Rico|1996-07-02	Puerto Rico	1996-07-02
GQ199884	GQ199884|Dengue_virus_4|4_II|Puerto Rico|1996-07-02	Puerto Rico	1996-07-02
GQ199882	GQ199882|Dengue_virus_4|4_II|Puerto Rico|1996-07-02	Puerto Rico	1996-07-02
FJ882595	FJ882595|Dengue_virus_4|4_II|Puerto Rico|1998-07-02	Puerto Rico	1998-07-02
FJ882596	FJ882596|Dengue_virus_4|4_II|Puerto Rico|1998-07-02	Puerto Rico	1998-07-02
FJ882601	FJ882601|Dengue_virus_4|4_II|Puerto Rico|1999-07-02	Puerto Rico	1999-07-02
MF004387	MF004387|Dengue_virus_4|4_II|Senegal|1981-07-02	Senegal	1981-07-02
GQ398256	GQ398256|Dengue_virus_4|4_II|Singapore|2005-07-02	Singapore	2005-07-02
KY921909	KY921909|Dengue_virus_4|4_II|Singapore|2016-03-15	Singapore	2016-03-15
JX024758	JX024758|Dengue_virus_4|4_II|Singapore|2010-12-15	Singapore	2010-12-15
AY618991	AY618991|Dengue_virus_4|4|Thailand|1977-07-02	Thailand	1977-07-02
AY618993	AY618993|Dengue_virus_4|4_II|Thailand|2000-07-02	Thailand	2000-07-02
FJ639736	FJ639736|Dengue_virus_4|4_II|Venezuela|1998-07-02	Venezuela	1998-07-02
FJ639744	FJ639744|Dengue_virus_4|4_II|Venezuela|1999-07-02	Venezuela	1999-07-02
FJ639739	FJ639739|Dengue_virus_4|4_II|Venezuela|1998-07-02	Venezuela	1998-07-02
FJ639742	FJ639742|Dengue_virus_4|4_II|Venezuela|1999-07-02	Venezuela	1999-07-02
FJ639745	FJ639745|Dengue_virus_4|4_II|Venezuela|1999-07-02	Venezuela	1999-07-02
FJ639748	FJ639748|Dengue_virus_4|4_II|Venezuela|2000-07-02	Venezuela	2000-07-02
FJ639738	FJ639738|Dengue_virus_4|4_II|Venezuela|1998-07-02	Venezuela	1998-07-02
FJ639764	FJ639764|Dengue_virus_4|4_II|Venezuela|2001-07-02	Venezuela	2001-07-02

FJ639737	FJ639737|Dengue_virus_4|4_II|Venezuela|1998-07-02	Venezuela	1998-07-02
GQ868642	GQ868642|Dengue_virus_4|4_II|Venezuela|2007-07-02	Venezuela	2007-07-02
EU854299	EU854299|Dengue_virus_4|4_II|Venezuela|2007-07-02	Venezuela	2007-07-02
FJ882586	FJ882586|Dengue_virus_4|4_II|Venezuela|2007-07-02	Venezuela	2007-07-02
FJ182016	FJ182016|Dengue_virus_4|4_II|Venezuela|2007-07-02	Venezuela	2007-07-02
FJ882584	FJ882584|Dengue_virus_4|4_II|Venezuela|2007-07-02	Venezuela	2007-07-02
JN819406	JN819406|Dengue_virus_4|4_II|Venezuela|2006-07-02	Venezuela	2006-07-02
HQ332172	HQ332172|Dengue_virus_4|4_II|Venezuela|2007-07-02	Venezuela	2007-07-02
HQ332176	HQ332176|Dengue_virus_4|4_II|Venezuela|2007-07-02	Venezuela	2007-07-02
FJ882585	FJ882585|Dengue_virus_4|4_II|Venezuela|2007-07-02	Venezuela	2007-07-02
EU854301	EU854301|Dengue_virus_4|4_II|Venezuela|2007-07-02	Venezuela	2007-07-02
FJ882592	FJ882592|Dengue_virus_4|4_II|Venezuela|2008-07-02	Venezuela	2008-07-02
FJ882588	FJ882588|Dengue_virus_4|4_II|Venezuela|2007-07-02	Venezuela	2007-07-02
FJ850095	FJ850095|Dengue_virus_4|4_II|Venezuela|2000-07-02	Venezuela	2000-07-02
FJ639773	FJ639773|Dengue_virus_4|4_II|Venezuela|2001-07-02	Venezuela	2001-07-02
JN819409	JN819409|Dengue_virus_4|4_II|Venezuela|1998-07-02	Venezuela	1998-07-02
JF262781	JF262781|Dengue_virus_4|4_II|Venezuela|1995-07-02	Venezuela	1995-07-02
JQ915090	JQ915090|Dengue_virus_4|4_II|Wallis_and_Futuna|2009-04-01	Wallis and Futuna	2009-04-01
